# READING and FEELING: the effects of a literature-based intervention designed to increase emotional competence in second and third graders

**DOI:** 10.3389/fpsyg.2014.01448

**Published:** 2014-12-16

**Authors:** Irina R. Kumschick, Luna Beck, Michael Eid, Georg Witte, Gisela Klann-Delius, Isabella Heuser, Rüdiger Steinlein, Winfried Menninghaus

**Affiliations:** ^1^Freie Universität BerlinBerlin, Germany; ^2^Humboldt-University BerlinBerlin, Germany; ^3^Charité University Hospital BerlinBerlin, Germany; ^4^Max-Planck-Institute for Empirical AestheticsFrankfurt am Main, Germany

**Keywords:** emotional competence, emotion understanding and knowledge, literature-based intervention, after-school care center, second and third graders

## Abstract

Emotional competence has an important influence on development in school. We hypothesized that reading and discussing children’s books with emotional content increases children’s emotional competence. To examine this assumption, we developed a literature-based intervention, named READING and FEELING, and tested it on 104 second and third graders in their after-school care center. Children who attended the same care center but did not participate in the emotion-centered literary program formed the control group (*n* = 104). Our goal was to promote emotional competence and to evaluate the effectiveness of the READING and FEELING program. Emotional competence variables were measured prior to the intervention and 9 weeks later, at the end of the program. Results revealed significant improvements in the *emotional vocabulary, explicit emotional knowledge,* and *recognition of masked feelings.* Regarding the treatment effect for detecting masked feelings, we found that boys benefited significantly more than girls. These findings underscore the assumption that children’s literature is an appropriate vehicle to support the development of emotional competence in middle childhood.

## INTRODUCTION

Over the past two decades, there has been an increasing interest in the importance of emotional competence not only in adults but also in children. Children with a high level of emotional competence are more able to regulate their feelings and are more successful at interacting with their peer group (Schultz et al., 2001; Smith, 2001; Trentacosta and Fine, 2010). They are not only quite popular among their classmates and make more friends but they also have better relationships with teachers (Ladd et al., 1999; Hamre and Pianta, 2001) and perform better academically (Gumora and Arsenio, 2002; Trentacosta and Izard, 2007; Denham et al., 2012). Thus, emotional competence is just as important as the improvement of cognitive and social skills.

Every individual will acquire various emotional skills in the course of his or her lifespan, thus becoming more and more emotionally competent. This paper uses the definition suggested by Saarni (1999, p. 5) who conceptualized emotional competence as a set of eight skills:

(1) Awareness of one’s emotional state, including the knowledge that it is possible to experience mixed feelings
(2) Ability to discern others’ emotions based on the knowledge about situational and expressive cues
(3) Ability to use the vocabulary of emotion and expression terms of one’s (sub)culture
(4) Capacity of empathic and sympathetic involvement in others’ emotional experience
(5) Understanding that inner emotional states do not need to correspond to outer expression, neither in oneself nor in others (masked feelings)
(6) Capacity for adaptive coping with aversive or distressing emotions by using self-regulatory strategies
(7) Awareness that the structure of relationships is largely defined by how emotions are communicated within the relationship
(8) Capacity for emotional self-efficacy

Saarni (1999) pointed out that these skills are not independent from each other because an increase in one skill can lead to gains in competence in one or more of the other skills. Like Denham (1998), she emphasized that each child has an emotional understanding and knowledge according to the child’s age which can be practiced and increased only within the framework of interpersonal situations. The quality of interaction with parents as well as the management of emotions in interaction with peers or teachers is decisively influenced by the child’s possession or lack of these skills. Thus, emotional and social competence are not entirely distinct: both follow overlapping developmental pathways and are complex, transactional, multifaceted constructs, consisting of a number of components (Rose-Krasnor, 1997; Saarni, 1999; Halberstadt et al., 2001) which are influenced by the continuous interplay between internal (e.g., ego identity, emotional self-efficacy) and external (e.g., school practices) factors (Humphrey et al., 2010).

The transition from kindergarten to elementary school can be regarded as an important milestone for social emotional development. With the start of kindergarten or school, the daily emotional challenges children have to face become more manifold, complex, and intensive. It is not only the case that children’s attachment needs with peers, (Salisch, 2001; Kerns et al., 2006) as well as with teachers, (Hamre and Pianta, 2001; Garner and Waajid, 2008) play an increasingly important role—the emotional lexicon, viewed as the comprehension and the use of mental states with an emotional dimension, also grows decisively in the time period from kindergarten to fifth grade (Baron-Cohen et al., 2010). Thus, it is especially important to foster emotional competence in elementary school, that means to train children explicitly in understanding how certain situations evoke specific emotions, in how to reflect one’s own and others’ emotional experience and in how to talk in an adequate way about them.

To improve emotional competence, intervention programs designed for children can play a major role. Nevertheless, Buckley et al. (2003, p. 11) criticize**:**

“Intervention programs most often emphasize broad social competencies as opposed to emotional competencies (…). For the most part existing assessment tools focus on three specific skills of emotional competence: emotional expression, empathy, and adaptive coping (see skills 3, 4, and 6). Less attention has been given to the remaining three skills: emotional awareness, understanding the emotions of others, and emotional dissemblance (see skills 1, 2, and 5). Given that the skills of emotional competence are reciprocally related, these omissions present a noteworthy limitation.”

Indeed, the socialization of emotional competence includes not only behavioral but also emotional understanding and knowledge aspects (i.e., knowledge about one’s own feeling, emotional dissemblance). The level of a child’s understanding of emotions or emotional knowledge is one essential source of individual differences that correlates with socioeconomic status in the peer group, attachment to teachers and school achievement (Izard et al., 2001, 2008; Miller et al., 2005). For instance, children with a low level of emotion knowledge are often rejected by peers, what in turn leads to withdrawal and, under vulnerable dispositions, to social anxiety (Schultz et al., 2001) or other, more externalizing, behavior problems (Speltz et al., 1999). In addition, Izard et al.’s (2001) path analysis identified emotion knowledge as a mediator of associations between verbal ability and academic competence. They argued that deficits in this ability, viewed as misperception or misinterpretation of emotion cues, contribute to negative behavioral outcomes and learning problems. Furthermore, to be aware of one’s own feelings and to acknowledge associated causes can be seen as a resilience factor in adolescence. For example, a teenager who is able to be aware of his or her own feeling of anger has a lower risk of drug abuse (Hessler and Katz, 2010). In sum, the knowledge and understanding aspects of emotional competence provides the foundation for emotional communication and social relationships with a long-term effect on motivational, psychological, occupational, and private development (Saarni, 1999; Izard et al., 2001; Trentacosta et al., 2006).

Furthermore, children in elementary school with lower emotional competence not only exhibit poorer academic development but they may also have less opportunity to improve their emotional skills, both at home and in the classroom. Therefore, a process that promotes emotional knowledge and understanding needs to be implemented within the framework of the peer group.

From the aspect of an adequate means to influence and enhance emotional competence, it is evident that a large body of stimulus material can be found in the field of children’s literature (Hogan, 2011). Through the reading of books which explicitly represent emotional experience through words, children gain not only in their cognitive abilities but also in their emotional knowledge (Beazidou et al., 2012). Obviously, both language abilities and the representation of emotional processes can be communicated via the framework of children’s literature (Hogan, 2011; Isbell et al., 2004; Wasik et al., 2006). Beazidou et al. (2012) indicated that, if teachers use the right literacy strategies, children’s books hold the potential to increase the specific emotional vocabulary (see skill 3). Moreover, storybook reading provides the opportunity to adopt several emotional perspectives of various characters and to engage children in emotional discourse (Hogan, 2011).

Language abilities, especially the presence of an emotion vocabulary, operate as a key factor in increasing emotional competence (Saarni, 1999, 2002). For instance, Troesch et al. (2012) showed that language skills predicted emotional knowledge in kindergarten children with an immigrant background. Indeed, a considerable amount of research revealed not only a significant relationship between language and emotional competence (Cutting and Dunn, 1999; Izard et al., 2001; Schultz et al., 2001; Harris and Pons, 2003; Pons et al., 2003; Bosacki and Moore, 2004; Beck et al., 2012), but also demonstrated that children with language impairment have limited abilities in several aspects of emotional competence (Redmond and Rice, 1998; Lindsay and Dockrell, 2000; Ford and Milosky, 2003; McCabe and Meller, 2004; Brinton et al., 2007; Nelson et al., 2011). This is in line with new research results that confirm a strong interrelation between various facets of emotional and language competences in middle childhood (Beck et al., 2012).

Altogether, it seems that language and literature are appropriate vehicles to develop emotional knowledge. Nevertheless, after thoroughly reviewing the existing literature we did not find any study that explicitly used the interactive reading of books to foster emotional competence in middle childhood. Hence, the major aim of the present study was to develop and evaluate a new intervention program on the basis of children’s literature aimed at improving emotional competence in children within the peer group setting. The development of adaptive and age-appropriate material and techniques was ensured by an interdisciplinary research team consisting of psychologists, linguists, psychiatrists, and literary scholars. Within the intervention program, the following aspects of emotional competence should be addressed: the understanding of emotions and emotional processes of others (in children’s literature), and the conscious perception and comprehension of emotional dissemblance and mixed feelings as well as emotional language in the story.

### DEVELOPMENT OF THE LITERATURE-BASED INTERVENTION READING AND FEELING

Particular attention was paid to the selection of the book. It needed to be age-appropriate, and it had to offer stimulus material for our subsequent work on the emotion-focused goals of the intervention. The book was selected in cooperation with specialists of children’s literature and children themselves. The final decision was made in favor of the book *Ein Schaf fürs Leben* (English title: Sheep with boots) written by the Dutch author Matter (2003). A literary analysis was conducted by a literary scholar in order to ensure the age-appropriateness of the book.

Certain characteristics of *Ein Schaf fürs Leben* (Matter, 2003), a story about a wolf and a sheep, were important for our decision to use it for our intervention: the emotional states and related thoughts, action tendencies, the physical body, and the facial expressions of boththe protagonist (the wolf) and the antagonist (the sheep)are described in great detail. In addition, the wolf displays a lot of masked and mixed feelings while the sheep is very emotionally direct and displays congruence between inner state and outer expressions. The book explicates behavioral tendencies and the regulation of an emotion using the example of anxiety. Thus, *Ein Schaf fürs Leben* provides its readers a stage for various forms of empathetic shifts in perspective and can be considered to be an adequate stimulus material.

The content of the book was divided into units to be read and discussed by the participants. The length of the intervention was set to eight sessions of 90 min each. In addition, the general and detailed aims for each of the eight units were specified. Finally, an intervention manual with detailed explanations (e.g., precisely formulated, open questions concerning the text) was developed. Each reading unit was discussed after a first draft by our interdisciplinary research team and evaluated in terms of helpful learning strategies and age-appropriateness before it was included as part of the manual. An overview of the eight units composing the READING and FEELING program is given in **Table 1**.

**Table 1:** Overview of eight lessons for the literature-based intervention READING and FEELING.

Topic	Aims	Diary of emotion tasks
1. Reading unit: The evaluation of an emotional stimulus from the perspective of the protagonist	Reflect on implicit prejudices related to the role models in the children’s book (sheep/wolf). When considering techniques of sensory perception, motor actions and introspective experience show that emotions are interactions between feeling, thinking, and behavior (e.g., embodied experiences with the verb “trotting”). Affective empathy: Empathize with the perspective of the protagonists and assign them appropriate emotions.	Semantic research project: Elaborate on the term “experiences” (a key concept in the book) through personal interviews with others and observation
2. Reading unit: The difference between true and masked feelings	Work out the different emotional motivations of the two protagonists for the example of the emotion “joy”: The sheep shows emotions directly/the wolf hides his intention. Work on the connection between the valence of emotions and the showing of emotions directly or masking them. Clarify the meaning of the term “experiences.” Understand that emotions can be recognized by describing movements, facial expressions, gestures, and breathing.	Emotion reporter: Interview other people about the topic “hiding/masking emotions”
3. Reading unit: Masked feelings	Introduction and development of the topic “masked feelings” by analyzing relevant text excerpts. Lying and body language: examine the indicators that make masked feelings readable. Narrative abilities: present progress of the story visually and continue telling the story with the help of a different illustration.	Emotion reporter: Interview people on “mixed feelings”
4. Reading unit: Mixed feelings	Introduction and development of the topic “Ambivalence of feelings/mixed feelings”: after playfully imitating the two opposite inner voices of the wolf, passages with two different emotions are analyzed to promote understanding of the connection between inner voices and emotions. The inner voices of wolf and sheep are hidden in the text. Those feelings have to be extracted from the text so that they become apparent.	Emotion reporter: Interview people about “fearful situations” and how they handle them
5. Reading unit: Inner voices of the characters and their feelings.	Closer look at the connection between inner voices and emotions, in picking up the inner phrases (aspects of the last unit), and physiological aspects of an emotion. Corresponding emotions are to be assigned to the inner voices. Individually select inner voices in the text. Experience that the inner voices in the new text unit elicit fear. As an example examine the feeling of fear: Recognize the sheep’s emotion, name it, and describe the four components of perception.	Emotion reporter: Collect emotion words for 1 week and write them down
6. Reading unit: Coping with anxiety and changeability of feelings	Understand that sheep and wolf change due to their (emotional) encounter. (Make reference to the stereotypical prejudices of the first reading unit.) Use the character of the wolf to show that emotions change. Bring up the wolf’s perception and coping strategy for fear based on the text.	Bring a stuffed animal or a pillow to the next meeting.
7. Reading unit: Memorization	Memorize by listening: The whole story is presented as an audio book. By this, the children have the opportunity of repeating the topics they have worked on so far.	
8. Reading unit: Applying the newly acquired knowledge to a different text.	“Emotion-in-text-task”: The children will apply their newly gained knowledge to an unfamiliar text during the reading exercise. Note: The children in the control group complete the same exercise.	

*The children completed one unit per week; one unit consisted of two subsequent lessons (45 min each). The diary of emotion was completed by the children between the different units.*

Each of the eight sessions included the introduction of the topic (by reading the selected text), a free discourse concerning the present topic, a structured group activity (often with body experiences, theater, or other creative techniques) and individual silent work (by using an emotion diary). During discourses concerning the selected text, the intervention instructors were encouraged to share their experiences and feelings with the group and, thus, assist as a role model in demonstrating appropriate emotional communication skills (Waliski and Carlson, 2008). Furthermore, the instructors were trained to support the group processes: with the help of open questions, they should enable each child to participate as a full member of the literature discussion group and to verbalize own thoughts about the text in order to learn that one’s own personal interpretation might differ from the interpretation of peers (Astington and Filippova, 2005; Certo et al., 2010).

Prior to the intervention, parents received a letter containing detailed information about the reading program and about how the acquired data would be utilized. According to government regulations, parental authorization was required for the free participation of all children in the READING and FEELING program (LDA Brandenburg, 2010). To support the aims of the intervention, we especially encouraged them to answer all questions raised by their children about emotions during the course of the 8-week program.

The development of the literature-based intervention aimed at increasing four language-related emotional competences: explicit emotional knowledge, emotional vocabulary, detection and appropriate labeling of mixed feelings, and recognition of masked feelings. Primarily, we wanted to know whether the READING and FEELING program is an appropriate vehicle to increase emotional competence in second and third graders. In addition, we sought to determine whether children had increased their capability of analyzing and understanding texts.

## MATERIALS AND METHODS

### DESIGN

The study was carried out in the form of a controlled quasi-experimental intervention and control group design (*n* = 208) with pre- and post-test assessment. An equal number of intervention and control group children were selected from ten after-school care centers. Children were asked to participate in the intervention or the control group as part of their afternoon care program. The control group took part in the regular day care program, i. e., the children were playing in groups instructed by an educator. The major aim of the evaluation study was to find out whether replacing the regular daycare program by a literature-based intervention would increase emotional competences. The educators were asked to assign the children to one of the two groups. Finally, 15 groups, each with seven children, participated in our intervention program during two consecutive weekly lessons (45 min per lesson) over a period of 8 weeks. All children (including the 104 in the control group) were tested before the first session of the intervention and directly after the last session of the intervention (in the following called “time t1 and t2”).

Prior to the program implementation, four intervention instructors were specially trained and prepared during a 2-day training session based on a standardized intervention manual (description and overview; see **Table 1**). The instructors then had the opportunity to practice teaching the lessons. All of the intervention instructors were aged between 30 and 40 years. In all but one case, they were students of psychology who had already successfully completed an apprenticeship in a corresponding professional area and, in some cases, they even possessed working experience in the educational field. Weekly supervision sessions with the intervention instructors were implemented to ensure the accuracy, quality, and consistency of the program across the 15 groups. Thus, any problem that arose could be addressed, discussed, and supervised in the supervision sessions.

### SUBJECT SELECTION AND DATA COLLECTION

Participants were recruited from 10 after-school childcare centers in a small city in Brandenburg, which is a federal state of Germany. Brandenburg has a very low proportion of immigrants (2.6%). Consequently, ethnic minorities were severely underrepresented in the present sample of children. Ninety-eight percent of the participants were Caucasian, while four children were either Asian or African. A majority of the children (98.1%) were German-speaking (monolingual), while a minority of the participants was speaking another mother tongue such as Vietnamese or Lingala. Within each of the 10 after-school childcare centers, a minimum of two groups (an intervention group and a control group with seven children each) were created. In sum, 208 second and third grade children were selected for the study.

To determine the appropriate sample size, we consulted power tables for research designs where the assignment to the intervention or control group is not done on the level of individuals but on the level of groups. This was necessary because our literature-based intervention is a group-based program. We assumed a medium effect size of the intervention, a small intraclass correlation, and we determined that each group should consist of seven children because this group size seemed to be appropriate for implementing the literature-based intervention. According to power tables, 15 intervention groups of seven children each were needed (Barcikowski, 1981). Thus, 104 intervention group children (65 female; 30 7-year-olds, 50 8-year-olds, 24 9-year-olds) and 104 control group children (62 female; 31 7-year-olds, 55 8-year-olds, 18 9-year-olds) completed emotional competences tests before the intervention (t1). At time t1, the mean age in the literature-based intervention group was *M* = 7.94 (SD = 0.72) and in the control group, *M* = 7.91 (SD = 0.72).

Due to illness and relocation we experienced 6% attrition. Therefore, we conducted a logistic regression to analyze whether systematic dropouts existed in terms of sex, age, language, and emotional competence variables. Regarding the observed variables, there were no systematic dropouts at the post-test [χ^2^ (7) = 8.43, *p* = 0.30]. At time t2, the mean age in the intervention group was *M* = 8.13 (SD = 0.72) and in the control group, *M* = 8.06 (SD = 0.66).

### MEASURES

The present study assessed emotional and language competence as well as text analysis capability as follows:

Emotional competence variables, which we expected to change due to the intervention (emotional vocabulary, identification of masked feelings, explicit emotional knowledge, ability to discover mixed feelings), were assessed prior to (t1) and after the intervention (t2) with the help of a board game that was specifically developed for this purpose. In order to control language proficiency, multiple facets of language competence were measured at t1. Research assistants conducted direct assessments with children in the control group and intervention group in quiet locations at the after-school childcare centers. Each assessment involved a face-to-face interaction between the investigator and each tested child, which lasted for approximately 1½ h.

In addition, the text analysis capability of the children concerning different emotional aspects (performed only once at t2) was measured in the framework of a group test. Using a new text, the children were supposed to complete a reading exercise and apply the knowledge they had obtained throughout the course of the literature-based intervention. This *emotion-in-text-task* comprised the last session of the emotion-centered literary program. Control children also completed this task, and this assessment was carried out as a paper-and-pencil version suitable for group testing. The children worked independently on the emotion-in-text-task with limited response time.

### EMOTIONAL COMPETENCE VARIABLES

The *laboratory of feelings* is a board game consisting of five rooms drawn on the board through which the child must lead the figure of a Martian who does not know what emotions are. In each room the child has to solve a riddle or to answer questions about emotions in order to help the Martian understand the concept of emotions. This board game allowed the assessment of the following areas of emotional competence:

#### Emotional vocabulary

After being asked *Please name all of the different feelings you can think of*, children should list all the feelings they could think of (see also Kusche et al., unpublished). Children received one point for each correctly stated feeling but none for an incorrect response (e.g., the description of the behavior). The target variable was the number of correctly listed feelings (open–ended response format).

#### Explicit emotional knowledge

In response to a short sample situation (*e.g., “Imagine a boy, let’s call him Peter. Peter plays in a soccer game and he just scored the winning goal”*) we asked the children five questions concerning different components of an emotion (Scherer, 2001): (a) The conceptual attribution or label for the feeling in the given situation (How does Peter feel?), (b) Peter’s thoughts concerning that feeling, (c) the physiological reaction involved in that feeling, (d) the behavioral tendencies connected with that feeling, and (e) the facial expressions and body language that go along with that feeling. Sample situations were provided for five basic emotions: joy, sadness, surprise, fear, and anger. One point was given for each component answered correctly and none for an incorrect response. Thus, children were assigned a number of points ranging from 0 to 25 (Cronbach’s α pre-test = 0.75; α post-test = 0.75). All responses were assessed by two independent, trained raters. Interrater reliability was computed on 625 randomly selected answers (Cohen’s κ pre-test = 0.93; κ post-test = 0.91).

#### Recognition of masked feelings

After listening to a short story, the children had to recognize if somebody was masking or hiding his/her real feeling and to identify the true feeling. A multiple choice response scale with four categories was presented with the instruction to choose one answer. The scale consisted of six items presenting short stories according to which the children had to estimate the hidden feelings of two people. For example: *Peter comes home. His parents have been arguing repeatedly in recent months. “Is Daddy there?” he asks. His mother sighs and says in a husky voice. “Hmm ..., not tonight. No.” Then she claps her hands, smiles and shouts a bit too cheerfully: “To the table, rascal! I’ve made you a pancake!” Peter does not think it is a good sign if Dad is not there. He feels lonely and like he is being let down by the whole world but he smiles and responds: “Wow, delicious pancakes!” What feeling is Peter masking? What feeling is his mother masking?* (*Response possibilities:* joy, disgust, sadness, pride, anger). The task was considered to be correct only if the child correctly recognized the masked feelings of both people. (The items are homogeneous according to a one parameter logistic test model, model-based reliability estimates: pre-test:0.61; post-test:0.60).

#### Recognition of mixed feelings

After listening to a short story (presented on the computer), the children had to decide what the protagonist was feeling. A multiple choice item with four categories (two of them correct) was provided for each story. The children had to select the correct two feelings by pressing a computer button. The target variable was the number of correctly detected feelings. This scale covered seven situations. Item example: *imagine that you spent your vacation together with your beloved grandmother. Today is the day of your departure. Your grandmother brings you to the train station and at the last moment she gives you a present that you have been wanting for a long time. What are you feeling? Response possibilities: sadness, anger, jealousy, happiness, and/or disgust.* (The items are homogeneous according to a one parameter logistic test model, model-based reliability estimates: pre-test:0.61; post-test:0.63).

To avoid memory effects from the pre- to the post-test, parallel tests with different stories were developed for the recognition of masked and mixed feelings as well as for the knowledge about different dimensions of emotions. All new measures that were developed by the authors were checked for reliability, validity, and feasibility during preliminary studies.

### LANGUAGE COMPETENCE VARIABLES

In order to control for differences in language competence, a measure of general language abilities was constructed and used as a covariate in further analyses. In the study, the following facets of language competence were assessed.

#### Receptive vocabulary

To measure the receptive vocabulary, the German adaptation of the Peabody Picture Vocabulary Test (PPVT) from Dunn and Dunn (2004) was used. In this test the children had to match an image with the correct word. For each item four possible images were presented. This test consisted of 88 computer-based items. Internal consistency was α = 0.83. For each correct answer, one point was given.

#### Literacy

We assessed children’s reading comprehension by using the “Information comprehension test for primary schools,” (ELFE; Lenhard and Schneider, 2006). ELFE 1–6 is a reading comprehension test that is used for children from first to fifth grade. For our purposes we used the computer-based version of the test. Reading comprehension is assessed on the basis of the following aspects adding up to three subscales: comprehension of words, comprehension of phrases, and comprehension of texts (comprehensive reading). According to Lenhard and Schneider (2006), Cronbach’s α varies between α = 0.92 and α = 0.97.

#### Verbal fluency

To assess semantic-lexical skills, children were encouraged to list as many animals as possible in 60 s. No point was given for repetitions. The calculated interrater reliability of this test was Cohen’s κ = 0.90.

#### Narrative abilities

Within the scope of this test of narrative skills, children were encouraged to tell a story based on the textless picture book *A Boy, a Dog, a Frog and a Friend* (Mayer and Mayer, 1971). The children’s narratives were coded by two independent raters regarding the presence of 25 basic components (e.g., the introduction of the characters, fading of goal one, conflict theme one, resolution of conflict one, and so on). The interrater reliability computed on the basis of 20 randomly chosen narratives is Cohen’s κ = 0.87. The test has an internal consistency of α = 0.87. As another aspect of children’s narrative skills we assessed the applied evaluative devices (ED). ED form a heterogeneous group of interpretative, linguistic devices including explicit and implicit perspective marks. These perspective marks are used to make a story more exciting and to guide the attention of the listener. According to the categories of Reilly et al. (1990) and Bamberg and Damrad-Frye (1991), the following linguistic marks were coded by two independent raters: mental states (e.g., to think, to know, to remember, to believe), causality (why, because), verbal marks of being unsure (e.g., maybe), onomatopoeic telling (e.g., the dog barks: “woof woof”), and negation (something is marked by a negation: e.g., He had *no* parents). The computed interrater reliability of 20 randomly chosen narratives was Cohen’s κ = 0.90.

A factor analysis showed that these facets measure a general factor of language abilities (Beck et al., 2012). Thus, a language factor score for every child was calculated by computing an intial principal component analysis.

### TEXT ANALYSIS CAPABILITY

#### Emotion-in-text-task

To test the increase in children’s general analysis capability of different emotional aspects in children’s literature, we conducted an emotion-in-text-task (this was also carried out with the children in the control group). Each child had to stop at five tables situated in a room. At each table the child was required to answer emotion-focused questions to a text excerpt. Before beginning this test, the test instructor determined that each child was capable of understanding the underlying text. This was important because all tasks were time limited and based on five different text excerpts from one and the same underlying text. Furthermore, all tasks (the children completed in rotation) were related to issues that had been processed in the emotion-centered literary program. Thus, children had to recognize mixed and masked feelings, to locate and detect emotional words on the basis of another text excerpt, and to mark the components of an emotion (cognitive, physiological, expression, and behavior tendencies). Furthermore, they had to label a feeling after reading the description of an emotional situation. The reliability of this scale was α = 0.64.

### DATA ANALYSES

**FIGURE 1:**
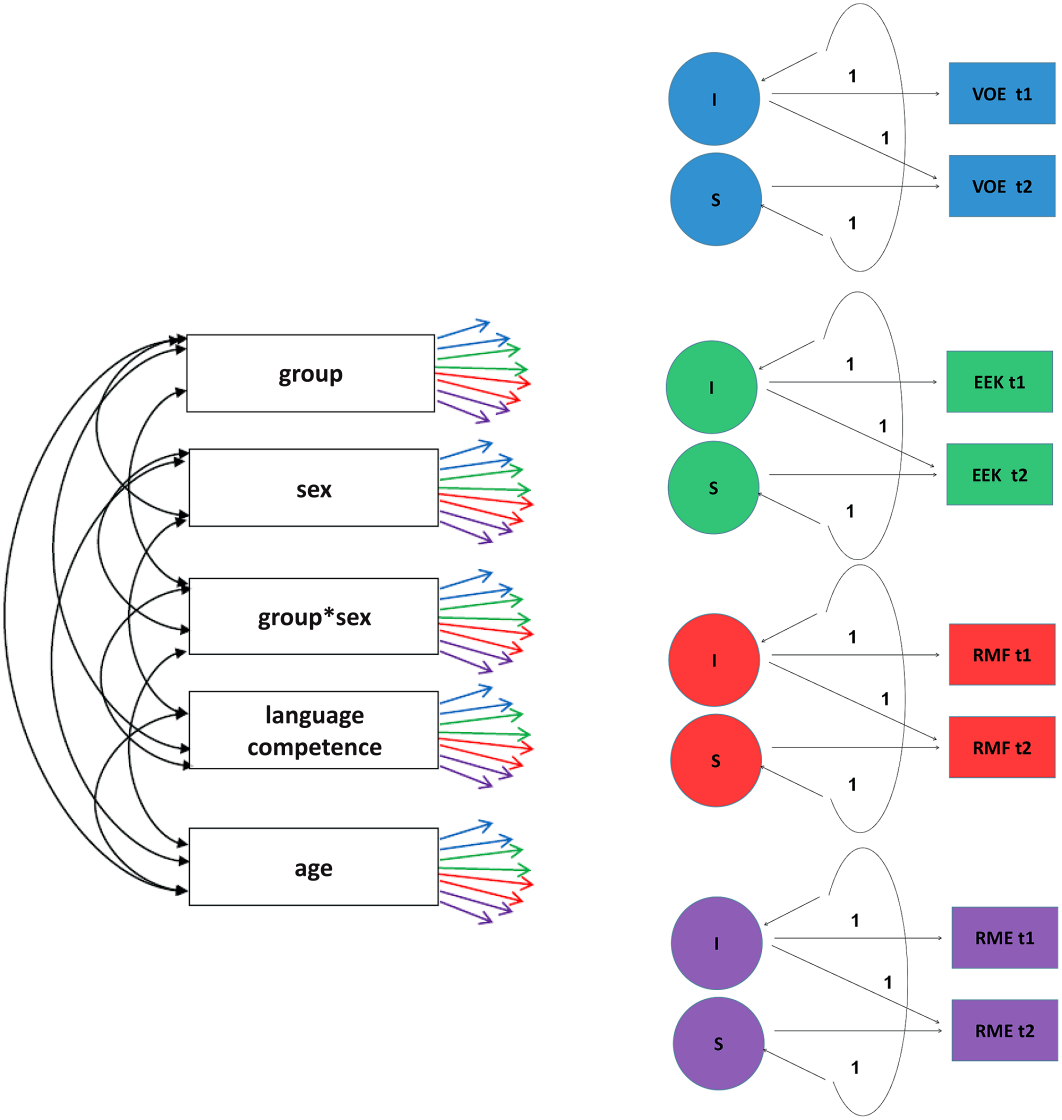
**Multivariate, manifest change-model.** The correlations between the residuals of the various facets of emotional competence have been omitted in this figure for better readability. VOE: Emotional Vocabulary; EEK: Explicit emotional knowledge; RMG: Recognition of masked feelings; RME: Recognition of mixed emotions; t1: time t1; t2: time t2; I: Intercept; S: Slope. The colored arrows indicate the regressive dependencies.

To test the effects of the literature-based program, a multivariate, manifest Change-Model was conducted. The analysis was performed with the computer program Mplus (Muthén and Muthén, 2010). Since the children were educated in small groups we had to include the multilevel structure in the database by selecting the MLR Complex estimation method. Thus, a cluster variable was incorporated in the model for the small group membership. Furthermore, the missing values were taken into account by the use of the Mplus full-information maximum likelihood estimator. The model is displayed in **Figure 1**.

The independent variables were language competence, age, group (intervention versus control), sex, and the interaction of the latter two. The correlations between the five independent variables were permitted. As already mentioned, for language competence we performed a confirmatory factor analysis (CFA) including the variables of receptive vocabulary, literacy, verbal fluency, and narrative abilities (Beck et al., 2012). The age variable was centered.

The dependent variables were emotional vocabulary, explicit emotional knowledge, recognition of masked feelings, and recognition of mixed feelings. In the multivariate Change-Model, the difference values for all four variables of emotional competence were defined so that for each criterion variable an intercept and a slope could be estimated. The correlations between the residuals of the criterion variables were permitted—also across the various facets of emotional competence.

For the analysis of the emotion-in-text-task, we conducted an ANCOVA with the between-subject factor group (control/intervention) and the control variables language competence and age.

## RESULTS

### RELATIONSHIPS BETWEEN THE INDEPENDENT VARIABLES

Correlations of the five independent variables as well as means and SDs are depicted in **Table 2**. We found a significant positive relationship between language competence and age (*r* = 0.22, *p* < 0.01). Moreover, the interaction of the group and sex variables correlated with the factor group (*r* = 0.67, *p* < 0.01) and with sex (*r* = 0.53, *p* < 0.01). Apart from that, there were no significant correlations. In addition, the descriptive statistics showed that the average of language competence (due to the principal component analysis) and age (due to centering) is zero.

**Table 2:** Correlations between the independent variables and descriptive Statistics.

	1	2	3	4	5
(1) Group	1				
(2) Sex	0.02	1			
(3) Group*Sex	0.67**	0.53**	1		
(4) Language competence	0.08	0.08	0.08	1	
(5) Age	0.02	0.01	-0.04	0.22**	1

*M*	0.50	0.61	0.31	0.02	-0.00
SD	0.50	0.49	0.46	4.29	0.71

n = 205, **p < 0.01 (two-tailed).

### EFFECTIVENESS OF THE LITERATURE-BASED INTERVENTION READING AND FEELING

Results of the Change-Model are presented in **Table 3**. The regression weights of intercepts revealed that no significant group differences existed at time t1. Prior to the intervention, the two groups had (in all four dependent variables) equal levels of emotional competence and did not differ by sex, (control or intervention) group and the interaction of both. In addition, we found significant correlations between language competence and each facet of emotional competence (*p* < 0.001) at time t1. Based on these results, we can conclude that the two groups were compatible prior to the intervention. Moreover, it became obvious that children with a higher level of language competence had an initial better test result in emotional competence.

The regression weights of slopes showed that the factor group significantly predicted the improvement in three facets of emotional competence to the benefit of the intervention group. Children in the intervention group reached a significant enhancement in their emotional vocabulary (*B* = 1.25, *p* < 0.001), explicit emotional knowledge (*B* = 1.32, *p* < 0.05) and recognition of masked feelings *(B* = 1.05, *p* < 0.001) compared to the children in the control group. It should be noted that the intervention effect with respect to the recognition of masked feelings was moderated by sex (interaction of sex and group: *B* = -1.14, *p* < 0.05). In terms of this variable, boys profited significantly more than girls. No significant treatment effect was found with respect to recognition of mixed feelings.

### GENERAL TEXT ANALYSIS CAPABILITY

We calculated the intraclass correlation coefficient (ICC) to test whether we had to take into account the clustering effect concerning the *Emotion-in-text-task-scale.* With an estimate of 0.001, the ICC was extremely low; therefore, it was not necessary to correct the *SE*s. To investigate if children of both groups differ significantly in their text analysis skills after the intervention, we calculated an ANCOVA with the between-subject factors group and sex, controlling for language competence and age. A partial eta-square of *η^2^* = 0.00 and *F*(1, 125) = 0.44 revealed no difference between the self-reliant text analysis skills of both groups (*p* = 0.51), but there was a gender effect in favor of girls concerning the *emotion-in-text-task* scale with *F*(1, 125) = 9.88, which was significant with a partial eta-square of *η^2^* = 0.08 (*p* < 0.01). However, no interaction was found between the factors group and sex [*F*(1,125) = 0.69, *p* = 0.42, *η^2^* = 0.01].

## DISCUSSION

The results of the current study generally support the assumption that a literature-based intervention increases emotional competences in children. The READING and FEELING program had a particularly beneficial effect on the enhancement of *emotional vocabulary* and *explicit emotional knowledge* of second and third graders. Furthermore, the effects of the program partly varied by gender of child. Thus, especially boys were positively influenced in their capability to *recognize masked feelings.* Overall, the current study confirms our hypothesis that children’s literature can be used as a model for analyzing everyday emotional processes and can consequently support emotional development.

Regarding the increase of emotional vocabulary and explicit emotional knowledge, we assume that the active, varied, and exciting approach contributed to the enhancement—particularly the variety of different learning techniques (Beazidou et al., 2012). As can be seen in **Table 1**, participants were engaged in several ways to highlight and discuss the different emotional perceptions of the story’s protagonists (e.g., the description of emotional expressions, interactions, related thoughts, and behavior in emotion-laden situations).

**Table 3:** Parameter estimates, standard errors and beta’s for the emotional competence measures.

	Intercept				Slope		
	*B*	SE	β		*B*	SE	β
**Emotional vocabulary**
Group	-0.10	0.43	-0.03		1.25***	0.36	0.28
Sex	-0.08	0.30	-0.02		1.09***	0.26	0.24
Sex*Group	-0.23	0.46	-0.06		-0.92	0.52	-0.19
Language competence	0.13***	0.03	0.30		-0.00	0.03	-0.01
Age	0.21	0.17	0.08		-0.46*	0.24	-0.15
**Explicit emotional knowledge**
Group	0.39	0.82	0.05		1.32*	0.74	0.19
Sex	0.34	0.43	0.04		1.11	0.58	0.15
Sex*Group	-0.14	0.89	-0.02		-1.52	0.95	-0.20
Language competence	0.44***	0.06	0.49		-0.11*	0.05	-0.13
Age	0.14	0.43	0.03		1.52	0.39	-0.02
**Recognition of masked feelings**
Group	-0.29	0.24	-0.12		1.05***	0.36	0.29
Sex	-0.18	0.21	-0.07		0.59	0.34	0.16
Sex*Group	0.17	0.32	0.07		-1.14*	0.49	-0.29
Language competence	0.07***	0.02	0.25		0.10**	0.03	0.23
Age	-0.25**	0.09	-0.14		-0.35**	0.14	-0.14
**Recognition of mixed feelings**
Group	0.09	0.28	0.03		0.25	0.35	0.08
Sex	0.36	0.29	0.11		0.20	0.42	0.06
Sex*Group	-0.00	0.37	-0.00		-0.55	0.48	-0.16
Language competence	0.15***	0.03	0.43		0.03	0.03	0.07
Age	0.20	0.14	0.09		-0.37*	0.17	-0.16

n = 205; control group = 0, intervention group = 1; male = 0, female = 1; *p < 0.05, **p < 0.01, ***p < 0.001.

The explicit emotional knowledge refers to the ability to understand an emotional experience as a complex occurrence consisting of different components (Scherer, 2001). To ensure the promotion of explicit emotional knowledge, we highlighted, discussed, and experienced the various components during program lessons in manifold ways. For example, children had the opportunity for body experiences: in order to better understand what the verb “trotting” in the story means—the children were supposed to imagine and then to act out what it looks like when the protagonist is trotting through the snow. With the involvement of further instructions such as “*What feeling(s) might be associated with this way of moving?*” and “*Is this a good or a bad feeling?,*” children learn (through conscious self-perception during motor actions) that the way of *moving* is directly connected to a feeling. Thus, the intervention promoted children’s active attempts to realize and understand the interaction between feeling, thinking, and behavior.

Moreover, the teachers were explicitly trained to act as role models by using elaborated words for feelings and explaining them and by giving information regarding emotions (Weare, 2000; Beazidou et al., 2012). Our intervention instructors were not only trained and encouraged to be role models by verbally expressing their emotions, but were also reminded on a weekly basis to explain feelings precisely. It might be that this verbal behavior of the teacher especially supported the enhancement of emotional vocabulary in children. In future studies, a collection of explained words or concepts for emotions would provide additional useful information.

In addition, results revealed a significant difference between participating and non-participating boys: boys in the treatment group especially improved their ability to recognize masked emotions, compared to the boys who were in the control group. How can we explain that this variable was affected most by the intervention in the group of boys? According to Saarni’s (2002) motivational categories, it is suggested that especially for boys in adolescence the ability to mask their feelings is a strategy to protect their self-esteem, to avoid negative consequences, and to manage their emotions according to the scripts of cultural display rules. Boys have to mask their feelings of anger and sadness within the peer group in order to avoid being teased (Zeman and Shipman, 1997). Therefore, it might be that, for boys, the knowledge about masked feelings is of special interest in order to prepare them for future developmental challenges such as adolescence (Brody and Hall, 1993; Zeman and Shipman, 1997). However, for boys, the aim of promoting the understanding and the use of hidden feelings in social interactions with the help of children’s literature has been achieved. Furthermore, the significantly higher score in the recognition of masked feelings could be due to the fact that the boys identified themselves more with the figure of the wolf in the book than the girls did.

The findings of the current study are limited in respect to two measured variables: no significant improvement could be detected in the recognition of mixed feelings as well as in the general ability to analyze literary texts. Altogether, it seems possible that second and third graders may simply have been too young to understand the complex concept of mixed feelings. Indeed, developmental research found that the ability to perceive and verbalize ambivalent feelings is only reliably available in later children’s development, at the age of 10 – 11 years (Salisch and Kunzmann, 2005). Another explanation for this finding may be that an 8-week literary intervention might be too short to perceptibly improve the ability to recognize and identify mixed feelings. Nevertheless, the program may have triggered important learning processes in terms of this variable with an effect that is detectable only later.

In addition, the results of the emotion-in-text-task indicated that the literature-based intervention did not noticeably increase the ability to deal with a new text excerpt in a text-analytic way. One reason might be that the techniques we used for explaining emotional phenomena during the intervention did not improve the literary analysis capacity in general. On the other hand, it is also possible that effectiveness with respect to this variable becomes evident only over time. Consequently, in future studies a follow-up assessment could provide insights into long term effects on the recognition of mixed feelings and the ability to analyze a literary text.

### FUTURE IMPLICATIONS AND DIRECTIONS

In summary, our findings indicate that our relatively short literary program is a feasible and promising intervention to stimulate greater understanding of emotions and emotion-related phenomena that was successfully implemented in 104 children in after-school care. The implementation of the literature-based intervention has shown that—contrary to behavioral-based approaches used in most interventions—a literary approach can influence children on a cognitive-affective level. This implies as well that the changes are not context specific. Rather, they form the foundations on which this new knowledge can be applied in a variety of different settings. Furthermore, we consider the greatest strengths of the present study to be found in the interdisciplinary approach (combining psychology and comparative literature) and the high degree of standardization of the methods and measures (e.g., the development of a manual with precisely formulated questions or the training and weekly supervision with the intervention instructors).

In conclusion, although further studies are required for analyzing the conditions under which emotional competence is best promoted in children, our present findings can be considered as an appeal for utilizing the multifaceted opportunities children’s literature provides in the school environment. Not only are early academic skills promoted by children’s literature, going beyond this, it also provides the potential to foster the theoretical education of emotional competence as well as social relationship skills through presentation in an emotion-focused way.

## Conflict of Interest Statement

The authors declare that the research was conducted in the absence of any commercial or financial relationships that could be construed as a potential conflict of interest.
